# Supplementary material for: Marketed Quinoa (Chenopodium quinoa Willd.) Seeds: A Mycotoxin-Free Matrix Contaminated by Mycotoxigenic Fungi
Source: Pathogens. 2023 Mar 6;12(3):418. doi: 10.3390/pathogens12030418 (PMC10057975; doi:10.3390/pathogens12030418)
Supplement: Supplementary file 1 [file pathogens-12-00418-s001.zip › pathogens-2224996-supplementary.pdf]

SUPPLEMENTARY MATERIAL

Table S1. Primers sequences, products sizes and annealing temperatures used in PCR assays for the identification of the different fungal genera associated with the marketed quinoa seed samples.

Locus	Primer	Sequences (5′ – 3′)	Product size (bp)	Annealing temp. (°C)	References
ACT	ACT-512F (Fw)	ATGTGCAAGGCCGGTTTCGC	300	61	41
	ACT-783R(Bw)	TACGAGTCCTTCTGGCCCAT			
BenA	Bt2a (Fw)	GGTAACCAAATCGGTGCTGCTTTC	550	55	42
	Bt2b (Bw)	ACCCTCAGTGTAGTGACCCTTGGC			
CaM	CMD5 (Fw)	CCGAGTACAAGGARGCCTTC	500	55	44
	CMD6 (Bw)	CCGATRGAGGTCATRACGTGG			
**ITS**	ITS1 (Fw)	TCCGTAGGTGAACCTGCGG	500	57	40
	ITS4 (Bw)	TCCTCCGCTTATTGATATGC			
RPB2	fRPB2- 5F (Fw)	GAYGAYMGWGATCAYTTYGG	1220	48-51	43
	fRPB2- 7cR(Bw)	CCCATRGCTTGYTTRCCCAT			
TEF1-α	EF1 (Fw)	ATGGGTAAGGA(A/G)GACAAGAC	700	53	36,38
	EF2 (Bw)	GGA(G/A)GTACCAGT(G/C)ATCATGTT			

Table S2. Cycling profiles used for the fungal genomic DNA amplification in the PCR assays.

Locus	1 step	35 steps*			1 step	References
	Initial denaturation	Denaturation	Annealing	Extension	Final extension	
ACT	95 °C x 4 min	94 °C x 45 s	60 °C x 40 s	72 °C x 1 min	72 °C x 10 min	41
BenA	95 °C x 4 min	94 °C x 1 min	67 °C x 1 min	72 °C x 1 min	72 °C x 10 min	45
CaM	95 °C x 4 min	95 °C x 1 min	55 °C x 1 min	72 ° C x 2 min	72 °C x 8 min	45
**ITS**	95 °C x 3 min	94 °C x 45 s	57 °C x 50 s	72 °C x 1 min	72 °C x 10 min	45
RPB2	95 °C x 5 min	95 °C x 1 min	47 °C x 1 min	72°C x 2 min	72 °C x 10 min	43
TEF1-α	94 °C x 5 min	94 °C x 60 s	53 °C x 1 min	72 °C x 1 min	72 °C x 10 min	36

*For *TEF1-α* gene amplification of *Fusarium* spp., 30 cycles of amplification were performed.

Table S3. *Alternaria* spp. isolates used in the phylogenetic analysis and related GenBank accession numbers.

Isolates[1]	Species	GenBank accession numbers	
		ITS	***RPB2***
CBS 534.83	*Alternaria abundans*	JN383485	KC584448
CBS 916.96[T]	*Alternaria alternata*	AF347031	KC584375
CBS 119396	*Alternaria alternarina*	KR425581	JQ905199
CBS 102605	*Alternaria arborescens*	NR135927	KC584377
CBS 210.86[T]	*Alternaria infectoria*	DQ323697	KC584404
CBS 115269	*Alternaria aspera*	KC584242	KC584474
AC90	*Alternaria atra*	LC440624	LC476833
CBS 200.67[T]	*Alternaria chartarum*	AF229488	KC584481
CBS 918.96	*Alternaria tenuissima*	AF347032	KC584435
Q 54	*Alternaria arborescens*	**OM892836**	**OM908530**
Q 113	*Alternaria alternata*	**OM892837**	**OM908531**
Q 132	*Alternaria abundans*	**OM892838**	**OM908532**
Q 149	*Alternaria infectoria*	**OM892839**	**OM908533**
Q 178	*Alternaria chartarum*	**OM892840**	**OM908534**
Q180	*Alternaria chartarum*	**OM892841**	**OM908535**
Q 184	*Alternaria alternata*	**OM892842**	**OM908536**
CBS 191.86[T]	*Stemphylum herbarum*	KC584239	KC584471

[1]CBS: Culture collection of the Centraalbureau voor Schimmelcultures, Fungal Biodiversity Centre, Utrecht, the Netherlands; Q: isolates obtained from the present study and deposited in the fungal culture collection of the Department of Agricultural, Food and Environmental Sciences, University of Perugia, Perugia, Italy. Bold type, GenBank accession numbers of these isolates. T= Type strain.

Table S4. *Penicillium* spp. isolates used in the phylogenetic analysis and related GenBank accession numbers.

**Isolates**[1]	**Species**	**GenBank accession numbers**	
		RPB2	***ITS***
**CBS 321.59**	*Penicillium aeneum*	KP064573	KP016812
**CBS 324.89**	*Penicillium aurantiogriseum*	JN406573	JN942751
**CBS 257.29**	*Penicillium brevicompactum*	JN406594	KF465776
**CBS 300.48**	*Penicillium canescens*	JN121485	MH856353
**F 727**	*Penicillium cellarum*	KM249117	KM249068
**CBS 126236**	*Penicillium chrystenseniae*	JN606624	MH863992
**CBS 306.48**	*Penicillium chrysogenum*	JN121487	MH856357
**CBS 321.59**	*Penicillium citreonigrum*	KP064573	MH857876
**CBS 139158**	*Penicillium citreosulfuratum*	KP064678	JX140939
**CBS 139162**	*Penicillium citreosulfuratum*	KP 064679	JX140940
**CBS 139.45**	*Penicillium citrinum*	JF417416	MH856132
**CBS 312.48**	*Penicillium corylophilum*	KP064631	MH856360
**CBS 115503**	*Penicillium crustosum*	MN969114	MH862985
**CBS 110412**	*Penicillium dipodomyis*	JX996474	MH862862
**CBS 325.48**	*Penicillium expansum*	JF417427	AB479309
**CBS 419.89**	*Penicillium flavigenum*	JF909939	MH862182
**CBS 101486**	*Penicillium freii*	JN606624	JN942735
**CBS 135.41**	*Penicillium hirsutum*	JN406629	MH856088
**CBS 221.28**	*Penicillium jaczewskii*	JN406612	MH854991
**CBS 161.81**	*Penicillium murcianum*	MN969202	MN431400
**5158**	*Penicillium nordicum*	KJ527378	KJ527448
**CBS 222.28**	*Penicillium polonicum*	JN985417	JN942711
**CBS 367.48**	*Penicillium restrictum*	JN121506	MH856396
**CBS 129667**	*Penicillium rubens*	JX996658	JX997057
**CBS 424.89**	*Penicillium solitum*	KU904363	MH860945
**PUMCH_Q141**	*Penicillium toxicarium*	MW122813	MT940755
**CBS 603.74**	*Penicillium verrucosum*	JN121539	AB479317
**CBS 390.48**	*Penicillium viridicatum*	JN121511	FJ613113
**Q 5**	*Penicillium verrucosum*	**OM908537**	**OM892850**
**Q 9**	*Penicillium chrysogenum*	**OM908538**	**OM892851**
**Q 35**	*Penicillium toxicarium*	**OM908540**	**OM892853**
**Q 39**	*Penicillium dipodomyis*	**OM908541**	**OM892854**
**Q 145**	*Penicillium polonicum*	**OM908542**	**OM892855**
**Q 181**	*Penicillium chrysogenum*	**OM908543**	**OM892856**
**CBS 310.38**	*Talaromyces flavus*	JF417426	JN899360

[1]CBS: Culture collection of the Centraalbureau voor Schimmelcultures, Fungal Biodiversity Centre, Utrecht, the Netherlands; Q: isolates obtained from the present study and deposited in the fungal culture collection of the Department of Agricultural, Food and Environmental Sciences, University of Perugia, Perugia, Italy. Bold type, GenBank accession numbers of these isolates.

Table S5. *Aspergillus* spp. isolates used in the phylogenetic analysis and related GenBank accession numbers.

Isolates[1]	Species	GenBank accession numbers	
		BenA	***CaM***
**CBS 557.65** T	*Aspergillus awamori*	AY820001	AJ964874
**CBS 101740** T	*Aspergillus brasiliensis*	AY820006	AM295175
**CBS 111.26** T	*Aspergillus carbonarius*	AY585532	AJ964873
**CBS 115574** T	*Aspergillus costaricaensis*	AY820014	EU163268
**DTO 321_G4**	*Aspergillus creber*	ON807807	ON807944
**NRRL 227** T	*Aspergillus cvjetkovicii*	EF652264	EF652352
**CBS 122712** T	*Aspergillus eucalypticola*	EU482435	EU482433
**NRRL 302** T	*Aspergillus flavipes*	EU014085	EF669549
**CBS 100927** T	*Aspergillus flavus*	EF661485	EF661508
**CBS 133.61** T	*Aspergillus fumigatus*	EF669791	EF669860
**NRRL 58600** T	*Aspergillus jensenii*	JN854007	JN854046
**NRRL225**	*Aspergillus jensenii*	JN854000	JN854020
**UTHSC 09-425**	*Aspergillus jensenii*	LN898858	LN898781
**CBS 589.65** T	*Aspergillus nidulans*	EF652251	EF652339
**CBS 554.65** T	*Aspergillus niger*	AY585536	AJ964872
**CBS 115.27** T	*Aspergillus niveus*	EF669528	EF669573
**CBS 108.08** T	*Aspergillus ochraceous*	EF661322	EF661381
**CBS 100926** T	*Aspergillus parasiticus*	EF661481	EF661516
**CBS 112811** T	*Aspergillus piperis*	AY20013	EU163267
**CBS 756.74** T	*Aspergillus pseudoflectus*	EF652331	EF652419
**CBS 593.65** T	*Aspergillus sydowii*	EF652274	EF652362
**CBS 103.14** T	*Aspergillus tamarii*	EF661474	EF661526
**CBS 134.48** T	*Aspergillus tubingensis*	AY820007	AJ964876
**CBS 113365** T	*Aspergillus vadensis*	AY585531	EU163269
**CBS 139.54** T	*Aspergillus welwitschiae*	FJ629291	KC480196
**Q 29**	*Aspergillus jensenii*	**OM974258**	**OM974262**
**Q 49**	*Aspergillus fumigatus*	**OM974259**	**OM974263**
**Q 73**	*Aspergillus tubingensis*	**OM974260**	**OM974264**
**Q 146**	*Aspergillus flavus*	**OM974261**	**0M974265**
**CBS 310.38** T	*Talaromyces flavus*	JX494302	KF741949

[1]CBS: Culture collection of the Centraalbureau voor Schimmelcultures, Fungal Biodiversity Centre, Utrecht, the Netherlands; NRRL: Mycological collection of the National Regional Research Laboratory, Peoria, IL, USA. Q: isolates obtained from the present study and deposited in the fungal culture collection of the Department of Agricultural, Food and Environmental Sciences, University of Perugia, Perugia, Italy. Bold type, GenBank accession numbers of these isolates. T= Type strain.

Table S6. *Cladosporium* spp. isolates used in the phylogenetic analysis and related GenBank accession numbers.

Isolates[1]	Species	GenBank accession number	
		ITS	*ACT*
**CBS 121624[T]**	*Cladosporium allicinum*	EF679350	EF679502
**CBS 125984[T]**	*Cladosporium australiense*	HM147999	*HM148486*
**CBS 112388[T]**	*Cladosporium cladosporioides*	HM148003	*HM148490*
**CPC 10142**	*Cladosporium cladosporioides*	HM148015	*HM148502*
**CBS 143361**	*Cladosporium. parasubtilissimum*	MF473170	MF474018
**CBS 125993**	*Cladosporium pseudocladosporioides*	HM148158	*HM148647*
**CBS 139572**	*Cladosporium uwebraunianum*	KP701873	KP701996
**CBS 121621[T]**	*Cladopsorium herbarum*	EF679363	EF679516
**Q 55**	*Cladosporium cladosporioides*	**OM892843**	**OM906941**
**Q 61**	*Cladosporium allicinum*	**OM892844**	**OM906942**
**Q 77**	*Cladosporium. parasubtilissimum*	**OM892845**	**OM906943**
**Q 92**	*Cladosporium pseudocladosporioides*	**OM892846**	**OM906944**
**Q 111**	*Cladosporium uwebraunianum*	**OM892847**	**OM906945**
**Q 131**	*Cladosporium pseudocladosporioides*	**OM892848**	**OM906946**
**Q 162**	*Cladosporium cladosporioides*	**OM892849**	**OM906947**
**CBS 116456**	*Cercospora beticola*	NR121315	AY840458

[1]CBS: Culture collection of the Centraalbureau voor Schimmelcultures, Fungal Biodiversity Centre, Utrecht, the Netherlands; CPC: Culture collection of Pedro Crous, housed at CBC; Q: isolates obtained from the present study and deposited in the fungal culture collection of the Department of Agricultural, Food and Environmental Sciences, University of Perugia, Perugia, Italy. Bold type, GenBank accession numbers of these isolates. T= Type strain.

Table S7. *Fusarium* spp. isolates used in the phylogenetic analysis and related GenBank accession numbers.

Isolates[1]	Phylogenetic species	Genbank accession numbers
		TEF1-α
**CBS 485.94**	*Fusarium acuminatum*	AB674279
**MAFF 239206**	*Fusarium avenaceum*	AB674293
**NRRL 25084**	FIESC 29-a	JF740715
**NRRL 52758**	FIESC 30-a	JF740833
**CBS 144134**	*Fusarium oxysporum*	MH485044
**CBS 146.95**	*Fusarium sambucinum*	KM231941
**CBS 393.93**	*Fusarium tricinctum*	AB674263
**CBS 100312**	*Fusarium verticillioides*	AB674288
**Q 185**	*Fusarium oxysporum*	**OM974256**
**CBS 125552**	*Geejayessia cicatricum*	HM626644
**OR 74A**	*Neurospora crassa*	XM959775

[1]CBS: Culture collection of the Centraalbureau voor Schimmelcultures, Fungal Biodiversity Centre, Utrecht, the Netherlands; MAFF: Central Bank of the micrmicrganisms section in the Ministry of Agriculture, Forestry and Fisheries GeneBank in Tsukuba, Japan; NRRL: Mycological collection of the National Regional Research Laboratory, Peoria, IL, USA. Q: isolate obtained from the present study and deposited in the fungal culture collection of the Department of Agricultural, Food and Environmental Sciences, University of Perugia, Perugia, Italy. Bold type, GenBank accession number of this isolate.

Table S8. *Alternaria*, *Aspergillus*, *Cladosporium*, *Fusarium* and *Penicillium* species reported in the literature at January 2023 on *Chenopodium quinoa*.

Fungal genus	Fungal species	Plant material	References
Alternaria	*Alternaria alternata*	root	48
		seed	52
	Alternaria infectoria	plant	50
Aspergillus	*Aspergillus flavus*	seed	18
	Aspergillius fumigatus	seed	18
	Aspergillus niger	seed	18
	Aspergillus oryzae	seed	18
	Aspergillu parasiticus	seed	18
	Aspergillus sidowii	seed	18
Penicilllium	*Penicillium aurantogriseum*	seed	18
	Penicilllium brevicompactum	seed, root	18,48
	Penicilllium canescens	seed	18
	Penicillium chrysogenum	seed	18
	Penicillium citrinum	seed	18
	Penicilllium commune	seed	18
	Penicilllium corylophilum	seed	18
	Penicillium crustosum	seed	18
	Penicillium griseofulvum	seed	18
	Penicillium hirsutum	seed	18
	Penicilllium jaczewskii	seed	18
	Penicillium murcianum	root	48
	Penicillium minioletum	root	48
	Penicilllium polonicum	seed	18
	Penicilllium solitum	seed	18
	Penicilllium viridicatum	seed	18
Fusarium	*Fusarium acuminatum*	plant	49
	Fusarium avenaceum	plant	49
	Fusarium brachygibbosum	seed	55
	Fusarium citri	seed	52
	Fusarium culmorum	plant	53
	Fusarium equiseti	seed	51
		plant	53
	Fusarium graminearum	plant	53
	Fusarium oxysporum	root	48
		plant	53
	Fusarium sambucinum	root	48
	Fiusaurium tricinctum	root	48
Cladosporium	*Cladosporium spp.*	seed	19,18
	Cladopsorium cladosporioides	panicle	54

Table S9. Average numbers of the fungal colonies of each genus or as total of the isolated fungi obtained from the 25 marked-bought quinoa seed samples.

Sampl	Metho	Total fungal gene	*Alternaria*	*Cladosporium*	*Penicillium*	*Fusarium*	*Aspergillus*	*Others fungal gener*
**1**	**TOTAL**	0,5 ± 0,186 abc	0 ± 1,46e-06 a	0,10 ± 5,96e-02 ab	0,10 ± 5,23e-02 ab	0 ± 1,46e-06 a	0 ± 2,05e-06 a	0,05 ± 3,40e-02 ab
**1**	**PDA**	0,1 ± 0,098 a B	0 ± 4,50e-06 a B	0 ± 4,81e-06 a B	0 ± 0,0804 a A	0 ± 0,0333 a A	0 ± 0,0648 a A	0,1 ± 0,0715 a A
**1**	**DFB**	0,9 ± 0,295 abc A	0,5 ± 1,33e-01 abc A	0,2 ± 9,09e-02 abc A	0,2 ± 0,0804 ab A	0 ± 0,0333 a A	0 ± 0,0648 a A	0 ± 0,0715 a A
**2**	**TOTAL**	0,25 ± 0,131 a	0 ± 1,46e-06 a	0,15 ± 7,30e-02 ab	0,05 ± 3,70e-02 a	0 ± 1,46e-06 a	0 ± 2,05e-06 a	0 ± 3,60e-06 a
**2**	**PDA**	0,1 ± 0,098 a A	0 ± 4,50e-06 a A	0,3 ± 4,81e-06 a A	0,1 ± 0,0804 ab A	0 ± 0,0333 a A	0 ± 0,0648 a A	0 ± 0,0715 a A
**2**	**DFB**	0,4 ± 0,197 ab A	0,1 ± 5,95e-02 ad A	0 ± 4,81e-06 a B	0 ± 0,0804 a A	0 ± 0,0333 a A	0 ± 0,0648 a A	0 ± 0,0715 a A
**3**	**TOTAL**	0,4 ± 0,167 ab	0 ± 1,46e-06 a	0,10 ± 5,96e-06 ab	0 ± 3,91e-06 a	0 ± 1,46e-06 a	0 ± 2,05e-06 a	0 ± 3,60e-06 a
**3**	**PDA**	0,2 ± 0,139 a A	0,2 ± 8,41e-02 abd A	0 ± 4,81e-06 a B	0 ± 0,0804 a A	0 ± 0,0333 a A	0 ± 0,0648 a A	0 ± 0,0715 a A
**3**	**DFB**	0,6 ± 0,241 ab A	0,4 ± 1,19e-01 abcd A	0,2 ± 4,81e-06 a A	0 ± 0,0804 a A	0 ± 0,0333 a A	0 ± 0,0648 a A	0 ± 0,0715 a A
**4**	**TOTAL**	1,8 ± 0,354 bcde	0 ± 1,46e-06 a	0 ± 7,35e-06 a	1,40 ± 1.96e-01 c	0 ± 1,46e-06 a	0 ± 2,05e-06 a	0,4 ± 9,62e-02 bc
**4**	**PDA**	0,6 ± 0,241 ab B	0 ± 4,50e-06 a A	0 ± 4,81e-06 a A	0,3 ± 0,0804 abc B	0 ± 0,0333 a A	0 ± 0,0648 a A	0,3 ± 0,0715 a B
**4**	**DFB**	3 ± 0,539 cde A	0 ± 4,50e-06 d A	0 ± 4,81e-06 a A	2,5 ± 0,0804 c A	0 ± 0,0333 a A	0 ± 0,0648 a A	0,5 ± 0,0715 b A
**5**	**TOTAL**	1,95 ± 0,368 cde	1,55 ± 1,20e-01 b	0,05 ± 4,21e-02 ab	0,00 ± 3,91e-02 a	1,55± 1,26e-01 b	0,2 ± 6,37e-02 ab	0 ± 3,60e-06 a
**5**	**PDA**	0,5 ± 0, 220 ab B	0 ± 4,50e-06 a B	0,1 ± 6,43e-02 a A	0 ± 0,0804 a A	0 ± 0,0333 a B	0,4 ± 0,0648 b B	0 ± 0,0715 a A
**5**	**DFB**	3,4 ± 0,573 de A	0,3 ± 1,03e-01 abcd A	0 ± 4,81e-06 a A	0 ± 0,0804 a A	3,1 ± 0,0333 b A	0 ± 0,0648 a A	0 ± 0,0715 a A
**6**	**TOTAL**	2,6 ± 0,425 d	1,40 ± 1,20e-01 b	0,40 ± 1,19e-01 ab	0,05 ± 3,70e-02 a	1,40± 1,20e-01 b	0,05 ± 3,19e-02 ab	0,55 ± 1,13e-01 c
**6**	**PDA**	0,3 ± 0,170 a B	0 ± 4,50e-06 a B	0,2 ± 9,09e-02 ab B	0,1 ± 0,0804 ab A	0 ± 0,0333 a B	0 ± 0,0648 a A	0 ± 0,0715 a B
**6**	**DFB**	4,9 ± 0,689 de A	0,3 ± 1,03e-01 abcd A	0,6 ± 1,57e-01 bc A	0 ± 0,0804 a A	2,8 ± 0,0333 c A	0,1 ± 0,0648 a A	1,1 ± 0,0715 c A
**7**	**TOTAL**	0,5 ± 0,186 abc	0 ± 1,46e-06 a	0,15 ± 7,30e-02 ab	0 ± 3,91e-06 a	0 ± 1,46e-06 a	0,2 ± 6,37e-02 ab	0 ± 3,60e-06 a
**7**	**PDA**	0,6 ± 0,241 ab A	0 ± 4,50e-06 a B	0,2 ± 9,09e-02 ab A	0 ± 0,0804 a A	0 ± 0,0333 a A	0,4 ± 0,0648 b B	0 ± 0,0715 a A
**7**	**DFB**	0,4 ± 0,197 ab A	0,3 ± 1,03e-01 abcd A	0,1 ± 6,43e-02 ab A	0 ± 0,0804 a A	0 ± 0,0333 a A	0 ± 0,0648 a A	0 ± 0,0715 a A
**8**	**TOTAL**	0,95 ± 0,257 abcde	0 ± 1,46e-06 a	0,30 ± 1,03e-01 ab	0,20 ± 7,39e-02 ab	0 ± 1,46e-06 a	0,10 ± 4,51e-02 ab	0,15 ± 5,89e-02 abc
**8**	**PDA**	1,2 ± 0,341 ab A	0,1 ± 5,95e-02 ab A	0,5 ± 1,44e-01 abc B	0,4 ± 0,0804 abc B	0 ± 0,0333 a A	0,2 ± 0,0648 ab B	0 ± 0,0715 a B
**8**	**DFB**	0,7 ± 0,260 ab A	0,3 ± 1,03e-01 abcd A	0,1 ± 6,43e-02 ab A	0 ± 0,0804 a A	0 ± 0,0333 a A	0 ± 0,0648 a A	0,3 ± 0,0715 ab A
**9**	**TOTAL**	0,3 ± 0,144 a	0 ± 1,46e-06 a	0 ± 7,35e-06 a	0,10 ± 5,23e-02 ab	0 ± 1,46e-06 a	0 ± 2,05e-06 a	0,20 ± 6,80e-02 abc
**9**	**PDA**	0,6 ± 0,241 03 ab B	0 ± 4,50e-06 a A	0 ± 4,81e-06 a A	0,2 ± 0,0804 bc A	0 ± 0,0333 a A	0 ± 0,0648 a A	0,4 ± 0,0715 a B
**9**	**DFB**	0 ± 0,001 a A	0 ± 4,50e-06 d A	0 ± 4,81e-06 a A	0 ± 0,0804 a A	0 ± 0,0333 a A	0 ± 0,0648 a A	0 ± 0,0715 a A
**10**	**TOTAL**	1,75 ± 0,349 bcde	0 ± 1,46e-06 a	0,35 ± 1,11e-06 ab	0,15 ± 6,40e-02 ab	0 ± 1,46e-06 a	0,2 ± 6,37e-02 ab	0,25 ± 7,60e-02 abc
**10**	**PDA**	0,4 ± 0,197 ab B	0 ± 4,50e-06 a B	0, 7 ± 1,70e-01 bc A	0,1 ± 0,0804ab A	0 ± 0,0333 a A	0,3 ± 0,0648 ab B	0 ± 0,0715 a B
**10**	**DFB**	3,1 ± 0,548 cde A	1,6 ± 2,38e-01 ef A	0 ± 4,81e-06 a B	0,2 ± 0,0804 ab A	0 ± 0,0333 a A	0,1 ± 0,0648 a A	0,5 ± 0,0715 b A
**11**	**TOTAL**	0,25 ± 0,132 a	0 ± 4,50e-06 a	0,10 ± 5,96e-02 ab	0 ± 3,91e-06 a	0 ± 1,46e-06 a	0,05 ± 3,19e-02 ab	0,10 ± 4,81e-02 ab
**11**	**PDA**	0 ± 0,001 a B	0 ± 4,50e-06 a A	0 ± 4,81e-06 a B	0 ± 0,0804 a A	0 ± 0,0333 a A	0 ± 0,0648 a A	0 ± 0,0715 a B
**11**	**DFB**	0,5 ± 0,220 ab A	0 ± 4,50e-06 d A	0,2 ± 9,09e-02 abc A	0 ± 0,0804 a A	0 ± 0,0333 a A	0,1 ± 0,0648 a A	0,2 ± 0,0715 ab A
**12**	**TOTAL**	0,4 ± 0,167 ab	0 ± 1,46e-06 a	0,05 ± 4,21e-02 ab	0,05 ± 3,70e-02 a	0 ± 1,46e-06 a	0 ± 2,05e-06 a	0 ±3,60e-06 a
**12**	**PDA**	0,1 ± 0,098 a B	0 ± 4,50e-06 a B	0 ± 4,81e-06 a A	0,1 ± 0,0804 ab A	0 ± 0,0333 a A	0 ± 0,0648 a A	0 ± 0,0715 a A
**12**	**DFB**	0,7 ± 0,260 ab A	0,6 ± 1,460e-01 abce A	0,1 ± 6,43e-02 ab A	0 ± 0,0804 a A	0 ± 0,0333 a A	0 ± 0,0648 a A	0 ± 0,0715 a A
**13**	**TOTAL**	0,95 ± 0,257 abcde	0 ± 1,46e-06 a	0,05 ± 4,21e-02 ab	0,55 ± 1,23e-01 b	0 ± 1,46e-06 a	0 ± 2,05e-06 a	0,15 ± 5,89e-02 abc
**13**	**PDA**	0,7 ± 0, 260 ab A	0,1 ± 5,95e-02 ab A	0 ± 4,81e-06 a A	0,6 ± 0,0804 c A	0 ± 0,0333 a A	0 ± 0,0648 a A	0 ± 0,0715 a B
**13**	**DFB**	1,2 ± 0, 341 abce A	0,3 ± 1,03e-01 abcd A	0,1 ± 6,43e-02 ab A	0,5 ± 0,0804 bd A	0 ± 0,0333 a A	0 ± 0,0648 a A	0,3 ± 0,0715 ab A
**14**	**TOTAL**	0,4 ± 0,167 ab	0 ± 1,46e-06 a	0,20 ± 8,43e-02 ab	0,05 ± 3,70e-02 a	0 ± 1,46e-06 a	0 ± 2,05e-06 a	0,05 ± 3,40e-02 ab
**14**	**PDA**	0,2 ± 0,139 a A	0 ± 4,50e-06 a B	0,1 ± 6,43e-02 a A	0,1 ± 0,0804 ab A	0 ± 0,0333 a A	0 ± 0,0648 a A	0 ± 0,0715 a A
**14**	**DFB**	0,6 ± 0,241 ab A	0,2 ± 8,41e-02 abd A	0,3 ± 1,11e-01 abc A	0 ± 0,0804 a A	0 ± 0,0333 a A	0 ± 0,0648 a A	0,1 ± 0,0715 a A
**15**	**TOTAL**	0,85 ± 0243 abcde	0,05 ± 2,27e-02 a	0,50 ± 1,33e-01 b	0,10 ± 5,23e-02 ab	0,05± 2,27e-02 a	0,05 ± 3,19e-02 ab	0,05 ± 3,40e-02 ab
**15**	**PDA**	0,2 ± 0, 139 a B	0 ± 4,50e-06 a B	0 ± 4,81e-06 a B	0,2 ± 0,0804 abc A	0 ± 0,0333 a B	0 ± 0,0648 a A	0 ± 0,0715 a A
**15**	**DFB**	1,5 ± 0,381 bce A	0,2 ± 8,41e-02 abd A	1,0 ± 2,03e-01 c A	0 ± 0,0804 a A	0,1 ± 0,0333 a A	0,1 ± 0,0648 a A	0,1 ± 0,0715 a A
**16**	**TOTAL**	0,75 ± 0,228 abce	0 ± 1,46e-06 a	0 ± 7,35e-06 a	0,35± 9,78e-02 ab	0 ± 1,46e-06 a	0,3 ± 7,81e-02 b	0 ± 3,60e-06 a
**16**	**PDA**	0,6 ± 0,241 ab A	0,1 ± 5,95e-02 ab A	0 ± 4,81e-06 a A	0 ± 0,0804 a B	0 ± 0,0333 a A	0,5 ± 0,0648 b B	0 ± 0,0715 a A
**16**	**DFB**	0,9 ± 0,295 abc A	0,1± 5,95e-02 ad A	0 ± 4,81e-06 a A	0,7 ± 0,0804 d A	0 ± 0,0333 a A	0,1 ± 0,0648 a A	0 ± 0,0715 a A
**17**	**TOTAL**	2,1 ± 0,382 de	0 ± 1,46e-06 a	0,50 ± 1,33e-01 b	0 ± 3,91e-06 a	0 ± 1,46e-06 a	0 ± 2,05e-06 a	0,10 ± 4,81e-02 ab
**17**	**PDA**	1 ± 0,311 ab B	0 ± 4,50e-06 a B	1,0 ± 2,03e-01 bc B	0 ± 0,0804 a A	0 ± 0,0333 a A	0 ± 0,0648 a A	0 ± 0,0715 a B
**17**	**DFB**	3,2 ± 0, 557 cde A	3,0± 3,26e-01 f A	0 ± 4,81e-06 a A	0 ± 0,0804 a A	0 ± 0,0333 a A	0 ± 0,0648 a A	0,2 ± 0,0715 ab A
**18**	**TOTAL**	0,6 ± 0,204 abce	0 ± 1,46e-06 a	0,15 ± 7,30e-02 ab	0 ± 3,91e-06 a	0 ± 1,46e-06 a	0 ± 2,05e-06 a	0 ± 3,60e-06 a
**18**	**PDA**	0,1 ± 0,098 a B	0 ± 4,50e-06 a B	0,1 ± 6,43e-02 a A	0 ± 0,0804 a A	0 ± 0,0333 a A	0 ± 0,0648 a A	0 ± 0,0715 a A
**18**	**DFB**	1,1 ± 0,326 abce A	0,9 ± 1,79e-01 bce A	0,2 ± 9,09e-02 abc A	0 ± 0,0804 a A	0 ± 0,0333 a A	0 ± 0,0648 a A	0 ± 0,0715 a A
**19**	**TOTAL**	0,6 ± 0,204 abce	0 ± 1,46e-06 a	0,20 ± 8,43e-02 ab	0 ± 3,91e-06 a	0 ± 1,46e-06 a	0 ± 2,05e-06 a	0,10 ± 4,81e-02 ab
**19**	**PDA**	0 ± 0,001 a B	0 ± 4,50e-06 a B	0 ± 4,81e-06 a B	0 ± 0,0804 a A	0 ± 0,0333 a A	0 ± 0,0648 a A	0 ± 0,0715 a A
**19**	**DFB**	1,2 ± 0,340 abce A	0,6 ± 1,460e-01 abce A	0,4 ± 1,29e-01 abc A	0 ± 0,0804 a A	0 ± 0,0333 a A	0 ± 0,0648 a A	0,2 ± 0,0715 ab B
**20**	**TOTAL**	1,2 ± 0,289 abcde	0 ± 1,46e-06 a	0,60 ± 1,46e-01 b	0,2 ± 7,39e-02 ab	0 ± 1,46e-06 a	0,10 ± 4,51e-02 ab	0 ± 3,60e-06 a
**20**	**PDA**	2,1 ± 0,451 b B	0,2 ± 8,41e-02 ab A	1,1 ± 2,13e-01 c B	0,4 ± 0,0804 abc B	0,2 ± 0,0333 b B	0,2 ± 0,0648 ab B	0 ± 0,0715 a A
**20**	**DFB**	0,3 ± 0,170 ab A	0,2 ± 8,41e-02 abd A	0,1 ± 6,43e-02 ab A	0 ± 0,0804 a A	0 ± 0,0333 a A	0 ± 0,0648 a A	0 ± 0,0715 a A
**21**	**TOTAL**	0,2 ± 0,118 a	0 ± 1,46e-06 a	0,15 ± 7,30e-02 ab	0,05 ± 3,70e-02 a	0,1± 3,21e-02 a	0 ± 2,05e-06 a	0 ± 3,60e-06 a
**21**	**PDA**	0,3 ± 0, 170 a A	0 ± 4,50e-06 a A	0,3 ± 1,11e-01 abc B	0 ± 0,0804 a A	0 ± 0,0333 a A	0 ± 0,0648 a A	0 ± 0,0715 a A
**21**	**DFB**	0,1 ± 0,984 ab A	0 ± 4,50e-06 d A	0 ± 4,81e-06 a A	0,1 ± 0,0804 ab A	0 ± 0,0333 a A	0 ± 0,0648 a A	0 ± 0,0715 a A
**22**	**TOTAL**	0,1 ± 0,083 a	0 ± 1,46e-06 a	0,10 ± 5,96e-02 ab	0 ± 3,91e-06 a	0 ± 1,46e-06 a	0 ± 2,05e-06 a	0 ± 3,60e-06 a
**22**	**PDA**	0,2 ±0,139 a A	0 ± 4,50e-06 a A	0,2 ± 9,09e-02 ab B	0 ± 0,0804 a A	0 ± 0,0333 a A	0 ± 0,0648 a A	0 ± 0,0715 a A
**22**	**DFB**	0 ± 0,001 a A	0 ± 4,50e-06 d A	0 ± 4,81e-06 a A	0 ± 0,0804 a A	0 ± 0,0333 a A	0 ± 0,0648 a A	0 ± 0,0715 a A
**23**	**TOTAL**	0,45 ± 0,177 ab	0 ± 1,46e-06 a	0,05 ± 4,21e-02 ab	0,05 ± 3,70e-02 a	0 ± 1,46e-06 a	0,05 ± 3,19e-02 ab	0,05 ± 3,40e-02 ab
**23**	**PDA**	0,2 ± 0,139 a A	0 ± 4,50e-06 a B	0,1 ± 6,43e-02 a A	0 ± 0,0804 a A	0 ± 0,0333 a A	0 ± 0,0648 a A	0,1 ± 0,0715 a A
**23**	**DFB**	0,7 ± 0,260 ab A	0,5 ± 1,33e-01 abc A	0 ± 4,81e-06 a A	0,1 ± 0,0804 ab A	0 ± 0,0333 a A	0,1 ± 0,0648 a A	0 ± 0,0715 a A
**24**	**TOTAL**	0,1 ± 0,084 a	0 ± 1,46e-06 a	0 ± 7,35e-06 a	0 ± 3,91e-06 a	0 ± 1,46e-06 a	0,05 ± 3,19e-02 ab	0 ± 3,60e-06 a
**24**	**PDA**	0 ± 0,001 a A	0 ± 4,50e-06 a A	0 ± 4,81e-06 a A	0 ± 0,0804 a A	0 ± 0,0333 a A	0 ± 0,0648 a A	0 ± 0,0715 a A
**24**	**DFB**	0,2 ± 0,139 ab A	0,1± 5,95e-02 ad A	0 ± 4,81e-06 a A	0 ± 0,0804 a A	0 ± 0,0333 a A	0,1 ± 0,0648 a A	0 ± 0,0715 a A
**25**	**TOTAL**	0,95 ± 0,257 abcde	0 ± 1,46e-06 a	0,05 ± 4,21e-02 ab	0 ± 3,91e-06 a	0 ± 1,46e-06 a	0 ± 2,05e-06 a	0 ± 3,60e-06 a
**25**	**PDA**	0,8 ± 0,278 ab A	0,7 ± 1,57e-01 b A	0,1 ± 6,43e-02 a A	0 ± 0,0804 a A	0 ± 0,0333 a A	0 ± 0,0648 a A	0 ± 0,0715 a A
**25**	**DFB**	1,1 ± 0,326 abce A	1,1± 1,97e-01 ce A	0 ± 4,81e-06 a A	0 ± 0,0804 a A	0 ± 0,0333 a A	0 ± 0,0648 a A	0 ± 0,0715 a A

Average numbers of fungal colonies obtained in total (TOTAL) or either by isolation on Potato Dextrose Agar (PDA) or deep-freezing blotter test (DFB) from each market-bought quinoa seed sample as identified by morphological features. For each sample, the TOTAL column represents the average (± SE) of the 20 analyzed plates, each containing 10 seeds, for a total of 200 seeds, PDA and DFB columns represent the average (± SE) of 10 analyzed plates, each containing 10 seeds, for a total of 100 seeds per method. In each column, lowercase letters indicate significant differences (p= 0.05, Tuckey's multiple range test) between samples inside each class (TOTAL, PDA or DFB) and uppercase letters indicate significant differences (p= 0.05, Tuckey's multiple range test) between the two different isolation methods (PDA or DFB) inside each seed sample.

Table S10. Results of BLAST analysis.

Genus	Isolates	Species resulting from the *BLAST* analysis of the amplified regions	
		ITS	*RPB2*
Alternaria	Q 54	***A. alstroemeriae, A. alternata, A. tenuissima, A. burnsii, A. brassicicola, A. brassicae, A. gaisen, A. arborescens, A. longipes.***	***A. arborescens***, *A. alternata, A. postmessia, A. tenuissima, A. burnsii, A. yaliinficiens, A. citricancri, A. longipes, A. tectorum, A. setosae, A. daucifolii, A. iridicola, A. maritima, A. gaisen.*
	Q 113	***A. alternata, A. angustiovoidea, A. tenuissima, A. compacta, A. brassicae, A. solani, A. alstroemeriae.***	***A. alternata, A. postmessia***, *A. tenuissima, A. arborescens, A. tectorum.*
	Q 132	***A. abundans***, *A. pobletensis, A. infectoria, A. maloroum, A. rosae.*	***A. abundans***, *A. armoraciae, A. breviramosa, A. hordeiaustralica, A. dactylidicola, A. incomplexa, A. metachromatica, A. arbusti, A. novae-zelandiae, A. viburni, A. conjuncta, A. triticimaculans, A. infectoria, A. rosae, A. kulundi, A. ventricosa, A. papavericola, A. solani, A. cetera, A. arbusti, A. citricancri.*
	Q 149	***A. infectoria, A. cerasidanica, A. conjuncta, A. hordeicola, A. murispora***, *A. japonica, A. hordeiaustralica, A. ventricosa.*	***A. infectoriae, A. alternarina***, *A. hordeicola, A. californica, A. humuli, A. hampshirensis, A. infectoria, A. slovaca, A. triticimaculans.*
	Q 178	***A. chartarum, A. alternata***, *A. aspera, A. brassicaeporri, A. multiformis, A. consortialis, A. terricola.*	***A. atra***, *A. arborescens, A. postmessia, A. solani, A. alternata, A. metachromatica, A. rosae, A. vaccariae, A. japonica, A. papavericola, A. ethzedia, A. incomplexa, A. arbusti, A. infectoriae, A. ventricosa, A. conjuncta, A. tenuissima, A. horediaustralica, A. viburni, A. zinniae, A. concatenata, A. solani, A. yaliinficiens, A. longipes, A. novae-zelandiae, A. aspera, A. blumeae, A. calendulae.*
	Q180	***A. aspera, A. alternata, A. chartarum***, *A. brassicae, A. alternata, A. sorghi, A. consortialis, A. terricola, A cantlous, A. brassicae, A. multiformis.*	***A. atra***, *A. arborescens, A. alternata, A. postmessia, A. solani, A. burnsii, A. rosae, A. vaccariae, A. papavericola, A. japonica, A. metachromatica, A. incomplexa, A. ethzedia, A. arbusti, A. infectoria, A. ventricosa, A. citricancri, A. zinniae.*
	Q 184	***A. alternata, A. angustiovoidea, A. tenuissima, A. compacta, A. brassicae, A. solani***.	***A. alternata, A. tenuissima, A. postmessia***, *A. tectorum, A. longipes, A. yaliinficiens, A. citricancri.*
Penicillium	Q 5	***P. verrucosum***, *P. allii, P. albocoremium, P. viridicatum, P. hordei, P. nordicum, P. thomii, P. noeochinulatum, P. christenseniae, P. poloniucm, P. solitum, P. aurantiogriseum, P. hirsutum, P. cordubense, P. lapidosum.*	***P. nordicum***, *P. thymicola, P. freii, P. solitum, P. venetum, P. verrucosum, P. sublectaticum, P. polonicum, P. speluncae, P. aurantiogriseum, P. echinulatum, P. discolor, P. melanoconidium, P. crustosum, P. viridicatum, P. commune, P. cavernicola, P. noeoechinulatum.*
	Q 9	***P. chrysogenum, P. crustosum, P. griseofulvum, P. rubens, P. aetiophicum, P. commune, P. allii-sativi, P. fimorum.***	***P. chrysogenum***, *P. glycyrrhizacola, P. rubens, P. vanluyki, P. alii-sativi.*
	Q 35	***P. aeneum, P. citreonigrum, P. citreosulfuratum, P. toxicarium, P. fundyense.***	***P. citreonigrum***, *P. fundyense, P. toxicarium, P. citreoviride, P. citreosulfuratum, P. restrictum.*
	Q 39	***P. dipodomys, P. chrysogenum, P. griseofulvum, P. granulatum***, *P. lanosum, P. flavigenum, P. nalgiovense, P. vinaceum, P. commune.*	***P. dipodomys***, *P. nalgiovense, P. glycirrhizicola, P. chrysogenum, P. flavigenum, P. confertum, P. expansum.*
	Q 145	***P. polonicum***, *P. aurantiogriseum, P. cellarum, P. freii, P. christenseniae, P. viridicatum, P. neoechinolatum, P. melanoconidium, P. cyclopium, P. camemberti, P.sublectaticum, P. mali-pumilae, P. tricolor, P. speluncae, P. venetum, P. radicicola, P. commune, P. caseifulfum, P. tulipae, P. camemberti, P. echinolatum, P. allii, P. biforme, P. discolor, P. thymicolae, P. solitum, P. psychrotrophicum.*	***P. polonicum***, *P. aurantiogriseum, P. cellarum, P. freii, P. christenseniae, P. viridicatum, P. neoechinulatum, P. melanoconidum, P. cyclopium, P. sublectaticum, P. mali-pumilae.*
	Q 181	***P. olsonii, P. granulatum, P. chrysogenum, P. camemberti, P. allii-sativi, P. griseofulvum, P. flavigenum, P. crustosum, P. rubens, P. aethiopicum.***	***P. chrysogenum,*** *P. rubens, P. glycyrrhizacola.*
**Genus**	**Isolates**	**Species resulting from the BLAST analysis of the amplified regions**	
		ITS	***ACT***
Cladosporium	Q 55	***C. pseudocladosporioides, C. westerdijkiae, C. cladosporioides, C. asperulatum, C. perangustum, C. allicinum, C. uwebraunianum, C. delicatulum, C. inversicolor, C.subuliforme, C. tenuissima, C. oryzae, C. montecillanum, C. phyllophilum, C. europaeum.***	***C. cladosporioides***, *C. anthropophilum.*
	Q 61	***C. allicinum, C. herbarum, C. ramotenellum, C. iridiscucumerinum***	***C. allicinum, C.*** *bruhnei.*

	Q 77	***C. allicinum, C. herbarum, C. parasubtilissimum, C. iridis, C. floccosum, C. sinuosum, C. macrocarpum.***	***C. parasubtilissimum***, *C. allicinum, C. herbarum, C. subtilissimum, C. bruhnei, C. antarcticum, C. versiforme.*
	Q 92	***C. pseudocladosporioides, C. cladosporioides, C. funiculosum, C. perangustum, C. rectoides, C. uwebraunianum, C. inversicolor, C. subuliforme, C. tenuissimum, C. westerdijkiae, C. phyllophilum, C. delicatulum.***	***C. pseudocladosporioides***, *C. cladosporioides, C. funiculosum, C. halotolerans.*
	Q 111	***C. pseudocladopsorioides, C. cladosporioides, C. funiculosum, C. perangustum, C. rectoides, C. westerdijkiae, C. asperulatum, C. allicinum, C. uwebraunianum, C. delicatulum, C. inversicolor, C. subuliforme, C. tenuissimum, C. phyllophilum, C. europaeum.***	***C. uwebraunianum, C. pseudocladopsorioides,*** *C. australiense, C. angustisporum, C. phaenocomae, C. gamsianum, C. needhamense.*
	Q 131	***C. pseudocladopsorioides, C. cladosporioides, C. westerdijkiae, C. asperulatum, C. perangustum, C. allicinum, C. uwebraunianum, C. delicatulum, C. inversicolor, C. subuliforme, C. tenuissimum, C. montecillanum, C. europaeum.***	***C. pseudocladosporioides***, *C. cladosporioides, C. funiculosum, C. halotolerans, C. endoviticola.*
Cladosporium	Q 162	***C. pseudocladosporioides, C. cladosporioides, C. funiculosum, C. perangustum, C. rectoides, C. westerdijkiae, C. asperulatum, C. allicinum, C. uwebraunianum, C. delicatulum, C. inversicolor, C. tenuissimum, C. oryzae, C. phyllophilum.***	***C. cladosporioides***, *C. anthropophilum.*
**Genus**	**Isolates**	**Species resulting from the BLAST analysis of the amplified regions**	
		BenA	***CaM***
Aspergillus	Q 29	***A. jensenii, A. creber,*** *A. cvjetkovicii*	***A. jensenii, A. creber, A. cvjetkovicii***.
	Q 49	***A. fumigatus***	***A. fumigatus***
	Q 73	***A. tubingensis***	***A. tubingensis, A. niger, A. phoenicis***
	Q 146	***A. flavus***	***A. flavus***
**Genus**	**Isolates**	**Species resulting from the BLAST analysis of the amplified regions**	
		TEF1-a	
Fusarium	Q 185	***F. oxysporum***	

Bold type: species that gave the maximum similarity with the blasted sequence. **Gray highlighter**: species confirmed by the subsequent phylogenetic analysis

Table S11. Secondary metabolites detected in the freeze-dried culture of the isolates Q 146 of *Aspergillus flavus* obtained from marketed quinoa (*Chenopodium quinoa* Willd.) seed samples and grown on Czapek Yeast Autolysate (CYA) Agar medium.

***Aspergillus* secondary metabolites**	**μg Kg^{-1}**
**Aflatoxins**	
Aflatoxicol	551
Aflatoxin B1	64200
Aflatoxin B2	4860
Aflatoxin M1	1500
Aflatoxin P1	114
O-Methyl sterigmatocystin	367
Methoxysterigmatocystin	1
Sterigmatocystin	35.3
**Anthraquinoids**	
Averantin	435
Averufin	520
Norsolorinic acid	143
**Dihydrobenzofuran derivative**	
Asperfuran	632
**Diketopiperazines**	
Brevianimide F	9170
Cyclo (L-Pro_L-Tyr)	41700
Cyclo (L-Pro_L-Val)	19700
**Koningic acid**	
Heptelidic acid	202000
**Propionic acids**	
3-Nitropropionic acid	1970000
**Pyrones**	
Kojic acid	92800
**Sesquiterpenes**	
Sporogea AO	7130
**Versicolorin**	
Versicolorin A	262
Versicolorin C	919
Versiconal Acetate	51.6
Versiconol	4020
